# An Investigation of the Influence of Paste’s Rheological Characteristics on the Tensile Creep of HVFAC at Early Ages

**DOI:** 10.3390/ma18020305

**Published:** 2025-01-11

**Authors:** Tongyuan Ni, Kang Chen, Fangshi Gao, Xingrui Li, Yang Yang, Deyu Kong, Shuifeng Yao

**Affiliations:** 1 College of Civil Engineering, Zhejiang University of Technology, Hangzhou 310023, China; 211122060011@zjut.edu.cn (K.C.); 211123060045@zjut.edu.cn (F.G.); 211124060034@zjut.edu.cn (X.L.); kongdeyu@zjut.edu.cn (D.K.); 2112106031@zjut.edu.cn (S.Y.); 2 Key Laboratory of Civil Engineering Structures & Disaster Prevention and Mitigation Technology of Zhejiang Province, Hangzhou 310023, China

**Keywords:** nanoindentation, tensile creep, rheological properties of paste, fly ash, ZC model

## Abstract

The rheological properties of concrete paste significantly influence its tensile creep behavior. In this study, the tensile creep behavior of high-volume fly ash concrete (HVFAC) employing the same cementitious pastes was experimentally investigated, and the rheological properties of the paste containing a high volume of fly ash using the *nanoindentation* (NI) technique was investigated in order to explore the influence of the paste’s rheological properties (such as micro-mechanical properties and microscopic creep) on the early-age tensile creep of HVFAC. The results demonstrated that the micro-strain of paste containing a high volume of fly ash (HVFA) showed a larger value than that without fly ash. As the test age extends, a decreasing trend in microscopic creep was observed which could be attributed to the growth of the content of HD C–S–H (high density C–S–H) gel. Moreover, within the same age period, the experimental data revealed that the incorporation of fly ash resulted in the reduction of the values of the creep modulus ***C*** and characteristic time ***τ***. The effects of fly ash dosages and loading age on the creep properties of concrete was consistent with the micro-creep properties of the cementitious paste. The tensile specific creep values derived from the ZC (“ZC” are initials for the word ‘‘self-developed” in Chinese) model based on nanoindentation data closely match those obtained from experiments.

## 1. Introduction

**Figure 1:**
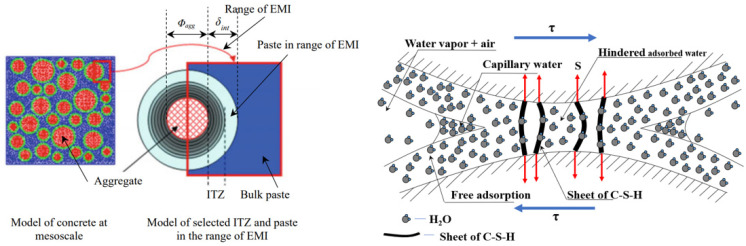
Schematic diagram of the C–S–H gel in the paste and the ITZ.

Creep can be beneficial or detrimental for a concrete structure, the fundamental mechanism of which has not been fully understood due to the complex microstructures of cementitious materials [1,2,3,4,5]. The creep of concrete has been widely studied for more than 100 years, and many prediction models of creep have been developed, such as the ACI code [6], the FIB model code [7], the JSCE code [8], the ZC model code [9], etc. Various physical phenomena have been investigated as potential origins of the basic creep of concrete and accounted for these models. It is possible that actual creep involves two or more mechanisms [10]. The concrete is a kind of heterogeneous materials, and the cement paste of that has an important influence on the viscoelastic behavior of concrete, especially for concrete under load. The aggregate was banded by hydration products of paste, and the interfacial transition zone (ITZ) around the aggregate, and the schematic diagram can be seen in Figure 1 [11]. The main source of the viscoelastic behavior of concrete in the microscale is the rheological properties of the C–S–H gel and ITZ [12,13]. On the other hand, fly ash (FA) is often used as a kind of supplementary cementitious material to improve the crack resistance and durability of concrete [14,15]. It has a significant influence not only the hydration products and hydration process of concrete directly and significantly but also influences the rheological properties of the C–S–H gel and ITZ, especially with ***high-volume fly ash concrete*** (HVFAC) [16,17,18,19]. Moreover, when the existing models are used to evaluate the creep of HVFAC, the model parameters need further exploration according the rheological properties of paste. It is important work to ascertain the role of paste’s rheological properties in HVFAC in order to predict the creep of HVFAC at early ages.

Moreover, most investigation about the tensile creep of fly ash concrete typically focuses on conventional fly ash dosages [20,21,22,23,24]. HVFAC, which experiences significant alterations in its hydration process and internal structure, exhibits pronounced variability in performance characteristics compared to normal dosage fly ash concrete, especially for the C–S–H gel structure and ITZ characteristics. It will influence the strength and stiffness development of the C–S–H gel within the paste and will play a pivotal role in concrete’s creep behavior [25,26]. The inclusion of substantial fly ash quantities directly impacts the temporal evolution pattern of C–S–H gel strength and stiffness in the hardened cement paste, subsequently affecting the mechanical properties of the concrete [18,27,28,29]. The volume content of the hardening cement pastes changes with the development of hydration and microstructures, and the volume content of the low-density and the high-density C–S–H gel also changes accordingly [30]. In recent years, the nanoindentation (NI) techniques have been applied to analyze the volume content of low-density and high-density C–S–H gels of hardened cement pastes [26,31,32]. NI techniques provide insights into the micro-mechanical behavior, deformation characteristics, and the physical phase distribution of C–S–H gels in cementitious materials [33,34,35]. The micromechanical properties of paste can be characterized using some parameters, such as hardness, microscopic creep, maximum indentation depth, cohesion coefficient, etc. [26,35,36,37,38,39]. Moreover, these parameters can be used to further clarify the role that the viscoelasticity of the paste plays in the creep of concrete, especially for HVFAC tensile creep at early ages.

In view of above facts, this study aims to investigate the role that the viscoelasticity of the paste plays in the HVFAC tensile creep at early ages and to examine the influence law of the paste’s rheological characteristic ontensile creep of HVFAC at the early age. The rheological properties of the paste containing high volume fly ash using NI techniques will be investigated in order to explore the influence of the paste’s rheological properties on the early-age tensile creep of HVFAC. Based on the experimental analysis of the paste’s nanoindentation and concrete tensile creep, the expressions of the ZC model considering the paste’s rheological characteristics, could be applied to estimate the development of HVFAC’s tensile creep.

## 2. The Fundamental Mechanisms of the ZC Model for Concrete Tensile Creep at Early Age

### 2.1. The ZC Model Based on Rheological Theory

Concrete is typically considered a material composed of three primary components: cement paste, ITZ, and aggregate [10,11]. The creep of concrete is commonly described as the slow and progressive deformation of concrete under a constant load, which is an intrinsic property of concrete materials [40,41]. Obviously, concrete creep is a behavior of viscoelastic materials, and it can be estimated by the creation of rheological models based on two fundamental elements: spring (Hooke body) and viscous dashpot (Newton body). The overall skeleton of the concrete is represented by the elastic unit member (spring) in the model. The elastic modulus in the elastic unit member is divided into the elastic modulus of the aggregate and the paste. The viscosity unit member (viscous dashpot) represents the paste and ITZ, and the essential cause of the creep is the deformation of the paste and ITZ.

**Figure 2:**
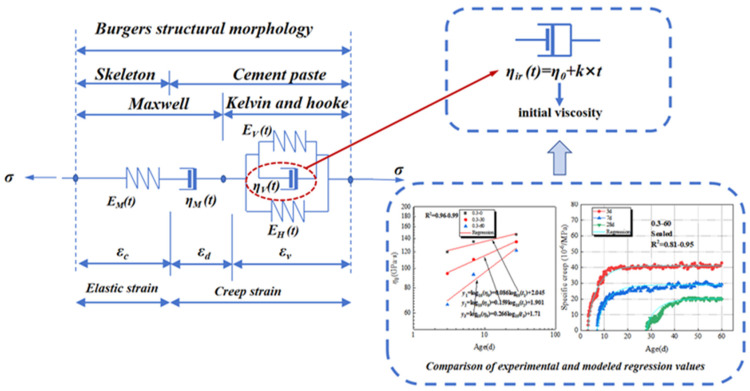
The micro-unit structure of the ZC model for the creep mechanism and the paste micro-creep of concrete.

Based on the reversibility assumption, Burgers’ rheological model assumes that the stiffness of hardened cement paste, formed after the hydration of cementitious material, changes from weak to strong. This change is considered the leading cause of variations in the rate of concrete creep. The structural morphology composition of Burgers model can be simulated and is shown in Figure 2. Burgers model is a simple combinate structural model used to describe and predict the concrete tensile creep, and the constitutive relations of the rheological components in Burgers model can be expressed by Equation (1).

(1) $$\sigma =E_{M}\varepsilon_{e}=\eta_{M}\dot{\varepsilon_{d}}=E_{V}\varepsilon_{d}+\eta_{V}\dot{\varepsilon_{V}}$$

The creep compliance function expression is represented in Equation (2):

(2) $$J(t,t_{0})=\frac{\varepsilon_{total}}{\sigma }=\frac{1}{E_{M}(t_{0})}+\frac{1}{\eta_{M}}(t-t_{0})+\frac{1}{E_{V}}\left\{1-\exp\left[-\frac{E_{V}}{\eta_{V}}(t-t_{0})\right]\right\}$$

The specific creep function expression is represented in Equation (3):

(3) $$C(t,t_{0})=\frac{1}{\eta_{M}}(t-t_{0})+\frac{1}{E_{V}}\left\{1-\exp\left[-\frac{E_{V}}{\eta_{V}}(t-t_{0})\right]\right\}$$

where $J(t,t_{0})$ = compliance function of concrete, $E_{M}$ = the elastic model of the Maxwell cell, $E_{V}$ = the elastic model of the Voigt cell, $\eta_{M}$ = the viscosity coefficient of the Maxwell cell, $\eta_{V}$ = the viscosity coefficient of the Voigt cell, $C(t,t_{0})$ = creep at age ***t*** due to a stress applied at age ***t_0_***.

The Burgers model was established based on the creep experiment of mature concrete, and the model parameters are only derived from the mathematical function and have no specific physical meaning. Yang [41,42,43] expounded on the mechanism of the degeneration of hardened cement paste components with the development of cementitious materials hydration by analyzing the relationship between different components produced by cement hydration and further clarified the physical significance of the model parameters. Basic on these, Yang [41,42] proposed the ZC (‘‘ZC” are initials for the word ‘‘self-developed” in Chinese) model after developing Burgers model. The structural morphology composition of the ZC model was introduced in the literature [44]. The specific explanation of the physical significance of the six model parameters was described in the literature, such as 1/χφ, φ, $1/(E_{V}+E_{H})$, $E_{H}/E_{V}$, q, r [41,42]. The physical meaning of parameter 1/χφ in the model is the specific creep of the Maxwell body when ***t*** → ∞, represented by $C_{M}(\infty ,t_{0})$; the physical meaning of parameter $1/(E_{V}+E_{H})$ in the ZC model is the specific creep of the Voigt body and the Hooke body when ***t*** → ∞, representation it by $C_{g}(\infty ,t_{0})$. The parameter q and r relate to the properties of the paste itself. Obviously, the paste’s viscoelasticity is closely connected to the production of cementitious materials through hydration. If the model parameters that reflect the paste’s viscoelasticity can be determined, the role that the paste’s viscoelasticity plays in concrete creep and its physical significance will become clearer.

Following the derivation of the ZC mode, the creep compliance and the specific creep function expression can be developed as follows:

(4) $$J(t,t_{0})=\frac{1}{E_{t}(t_{0})}+\frac{\left\{1-\exp[-\varphi (t-t_{0})]\right\}}{\chi \varphi }+\frac{1}{E_{V}+E_{H}}\{1-\exp[-(1+\frac{E_{H}}{E_{V}})\frac{{\left(t-t_{0}\right)}^{1-r}}{q(1-r)}]\}$$

(5) $$C(t,t_{0})=\frac{\left\{1-\exp[-\varphi (t-t_{0})]\right\}}{\chi \varphi }+\frac{1}{E_{V}+E_{H}}\{1-\exp[-(1+\frac{E_{H}}{E_{V}})\frac{{\left(t-t_{0}\right)}^{1-r}}{q(1-r)}]\}$$

The creep coefficient function expression can be represented as Equation (6).

(6) $$\phi (t,t_{0})=\frac{E_{t}(t_{0})}{\chi \varphi }\{1-\exp[-\varphi (t-t_{0})]\}+\frac{E_{t}(t_{0})}{E_{V}+E_{H}}\{1-\exp[-(1+\frac{E_{H}}{E_{V}})\frac{{\left(t-t_{0}\right)}^{1-r}}{q(1-r)}]\}$$

### 2.2. The Creep Mechanism of the ZC Model Considering the Paste’s Viscoelasticity

It is a major challenge to analyses the paste’s viscoelasticity during the hardening process in a microscopic way. In recent years, the nanoindentation (NI) is a good new technique to analyses the paste’s micro-characteristics, such as cement hydration products and micromechanical properties. and it can be further used to analyze the paste’s viscoelasticity. There may be differences in the paste’s viscoelastic properties under stressed or tensile states, and there was no technology able to detect the paste’s viscoelastic properties under tensile loading. Thus, the NI techniques was used to reflect the past viscoelasticity during the hardening process in a microscopic way in this study. According to the Oliver–Pharr methodology [38] and the principle of NI technology, the paste’s viscoelasticity can be reflected by several parameters, such as ***M*** (the indentation modulus), ***H*** (hardness), ***A_C_*, *L(t)-L(0)***, ***τ***, etc. [38,39] These parameters can be obtained with Equations (7)–(11).

(7) $$M=\frac{\sqrt{\pi S}}{2\beta \sqrt{A_{c}}}$$

(8) $$H=\frac{P_{max}}{A_{c}}$$

(9) $$A_{c}=\pi {\left(h_{c}tan\theta \right)}^{2}$$

(10) $$\frac{1}{M}=\frac{1-\upsilon^{2}}{E}=\frac{1-\nu_{i}^{2}}{E_{i}}$$

(11) $$L\left(t\right)-L\left(0\right)=\frac{2a_{c}∆h\left(t\right)}{P_{max}},\;L\left(0\right)=\frac{1}{M}$$

where, ***S*** is the contact stiffness, which is the slope of the initial stage of the unloading part of the ***P***-***h*** curve, S=dPdh; ***β*** is the correction coefficient of the indenter, ***β*** = 1.034; Ac is the projected contact area of the indenter; ***θ*** is the angle between the contact surface of the diamond indenter and the center line of the indenter; ***h***_c_ is the contact depth. The diamond indenter parameters used in this paper are as follows: the modulus of elasticity of the indenter ***E_i_*** = 1.141 GPa and Poisson’s ratio ***υ_i_*** = 0.07, where ***a_c_*** is the equivalent indentation radius of the contact projection area of the indenter.

Referring to the assumption of Vandamme M et al. [33], plastic deformation occurs only during the loading phase, while plastic deformation during the holding and unloading phases is small and is neglected in the calculation of contact creep. Under this assumption, the contact creep function can be expressed by Equation (11).

***∆h(t)*** is the change in indenter depth during the load-holding stage; *L(t)* is the contact creep flexibility at load-holding time ***t***.

According to the viscoelasticity theory [16], the viscous pot stress–strain relationship is expressed by Equation (12):

(12) $$\sigma_{0}=\eta_{ir}\frac{d\varepsilon }{dt}=(\eta_{0}+kt)\frac{d\varepsilon }{dt}$$

(13) $$C(t)=\frac{1}{k}\ln(1+\frac{t}{\eta_{0}/k})$$

(14) $$\left\{\begin{array}{l} k=C\\ \eta_{0}/k=\tau \end{array}\right.$$

The micro-unit structure of the ZC model that was created by our research team for the creep mechanism and the paste micro-creep of concrete was shown in the Figure 2. Obviously, the variability of the hydration process of the cement paste affects the development pattern of its maximum indentation depth ***h***_m_, hardness ***H*,** and other parameters directly. Thus, the time-dependent evolution of these parameters can provide a chance to analyze the rheological behavior of cementitious paste. The ZC model deduced from the rheological properties of the paste reflects the contribution of the cement paste to the development of the creep of concrete. Through the multivariate nonlinear regression method, he law depend on time of each parameter and the expression of ZC model about the development of early-age tensile creep of concrete can be obtained. This expression can reflect the micro-structure of the model unit and based on the rheological properties of the cementitious paste.

Based on the above, the specific creep function expression of the ZC model considering the paste viscoelasticity can be developed as follows:

(15) $$C\left(t,\;t_{0}\right)=\frac{1-\exp\left[-\varphi \left(t-t_{0}\right)\right]}{\chi \;\varphi }+\frac{1}{E_{V}+E_{H}}\left\{1-{\left[1+\frac{1}{\tau }\left(t-t_{0}\right)\right]}^{-\frac{E_{V}+E_{H}}{C}}\right\}$$

When targeting a specific concrete loading age, the cohesion coefficient $\eta_{ir}(t)=\eta_{0}+k(t-t_{0})$ of the clay pots model can be substituted into the cohesion coefficient $\eta_{V}$ in the ZC model in the form of $\left(t-t_{0}\right)$, and Equation (5) is obtained by solving the differential Equation (15).

Where $C(t,t_{0})$ denotes the specific creep of concrete and ***t_0_*** is the starting loading age; ***τ*** and ***C*** are the characteristics of time and contact creep modulus obtained from the nanoindentation experiments of the corresponding paste; $E_{H}$ and $E_{V}$ are the elasticity coefficients in the ***Hooke*** element and the elasticity coefficients in the ***Kelvin*** element.

## 3. Materials and Methods

### 3.1. Materials

The raw materials utilized in this study mainly include standard Portland cement of type P∙I 42.5 (in accordance with Chinese GB 175-2007) [45], fly ash that meets the grade II ash standard (in accordance with Chinese GB/T 1596-2017) [46], coarse and fine aggregates, tap water, and polycarboxylate superplasticizer with a water reduction ratio of 30% and a solid content ratio of 99%. The chemical compositions of the cement and fly ash are delineated in Table 1. The specific surface area of cement is 358 m^2^/kg, and the density of that is 3110 kg/m^3^. The density of fly ash is 2100 kg/m^3^. River sand with the fineness modulus of 2.8 was used as fine aggregate, and the apparent density of that is 2610 kg/m^3^. The crushed stone, consisting of 5–10 mm with apparent density of 2647 kg/m^3^, was used as coarse aggregate.

In this study, the principle of substituting cement with an equivalent mass of fly ash admixture in concrete was applied based on the reference mix ratio. The amount of polycarboxylate superplasticizer was adjusted to maintain the concrete slump at 180 mm ± 20 mm, and it met the requirements of pumping concrete according to the Chinese standard JGJ/T 10-2011 (Technical Specification for Concrete Pumping Construction) [47]. Additionally, to negate the impact of other variables, such as the water/binder ratio on concrete properties, the water/binder ratio, aggregate content, and sand rate were held constant while exploring the effects of fly ash admixture on concrete properties. In our test, concrete mixtures were set with a fixed water-to-binder (w/b) ratio of 0.3, an aggregate content is 0.65, and a sand rate is 0.36. The proportions of paste are shown in Table 2, and the proportions of HVFAC are shown in Table 3.

### 3.2. Methods of Experiments

#### 3.2.1. Mechanical Property of HVFAC

In this investigation, specimens with a size of 100 mm × 100 mm × 100 mm, which were matched in cross-sectional size with the creep specimens, were used for strength tests, and the specimens with a size of 100 mm × 100 mm × 400 mm were used for a tensile modulus of elasticity test. All of these specimens were demolded one day after concrete placement, enveloped in aluminum foil, and tested in a lab room in which the temperature and humidity were controlled and constant at 20 ± 1 °C and 60 ± 5%, respectively. The mechanical property of the HVFAC tests conducted at designated ages were executed according to Chinese standard GB/T 50081-2019 (Standard for Experimental Methods of Physical and Mechanical Properties of Concrete) [48]. Table 4 provides the detailed results from the HVFAC fundamental mechanical property tests average values. The compressive strength test values of all three samples varied within ±10% of the mean value, and the splitting tensile strength test values varied within ±7% of the mean value.

#### 3.2.2. Nanoindentation Experiments of Paste

As the hydration process of the cementitious materials progresses, the cementitious paste in concrete transitions from viscous and plastic states to viscoelastic and, eventually, to a cured state with a certain stiffness, contributing primarily to the volume deformation of early-age concrete [49]. To further explore the mechanisms of early-age tensile creep development in high-volume fly ash concrete and the impact of high fly ash content, the changing rheological characteristics of cementitious paste when a substantial quantity of fly ash was used to replace cement by equal mass would be investigated by nanoindentation technology in this study. And to examine the influence of fly ash and the intrinsic correlation between the contact creep function and the rheological parameters, the experiments of the HVFAC tensile creep and the paste micro-creep development in early age would be observed and analyzed. That is, the rheological parameters, such as the indentation modulus, hardness, ***A_C_***, ***L(t)-L(0)***, ***τ*** of paste with 0%, 30%, and 60% fly ash, were investigated.

The detailed procedure of the nanoindentation experiment is depicted in the literature [45]. Initially, the specimens were refined and polished using a UNIPOL-1200S apparatus (Shenyang Kejing Co. Ltd., Shenyang, China), followed by an assessment of surface roughness via the SuperViewW1 white light interferometer to confirm that the root-mean-square roughness of the specimen surfaces remained below 100 nm, aiming to minimize the experimental result variability. Thereafter, a designated area on each specimen’s surface was selected for conducting a 10 × 10 dot matrix indentation test with the Nano Indenter G200 (MML. English) equipment, applying a peak load of 2 mN at each test site and maintaining it for 180 s.

#### 3.2.3. Tensile Creep Experiment of HVFAC

The tensile creep test was executed employing a tensile creep measurement system designed by the Yang–Yang research team [9,19,28,41,42,43]. The methods of the shrinkage strains separated from the creep strains were introduced in the published literature [18] in the studies. The size of the tensile creep specimens and autogenous deformation specimens was 100 mm × 100 mm × 400 mm, and the experimental scene was shown in the literature [44], which introduced the initial results of this study. The resolution of the shrinkage strains and the tensile creep strains measured was 2.5 με. The ratio of stress to split strength was 0.4. The data of stresses and strains throughout the experiment was automatically logged by a collector (Type: TDS-530). In the test room, the temperature was 20 ± 1 °C, and the relative humidity was 60 ± 5% during the experiment period.

## 4. Results

### 4.1. The Paste Viscoelastic Behaviors

**Figure 3:**
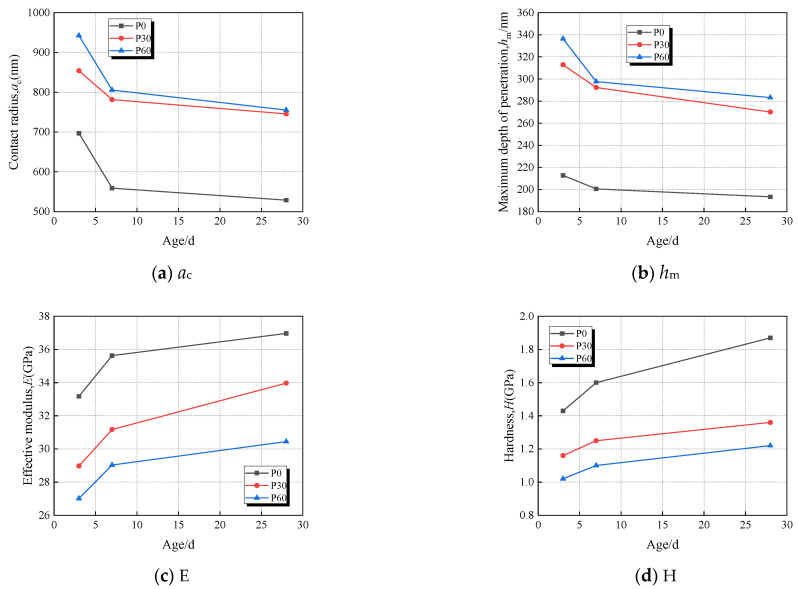
Mean changes in micromechanical properties of P0, P30, and P60 specimens. (**a**) Parameter *a*_c,_, (**b**) Maximum indentation depth ***h***_m_, (**c**) Effective modulus E, (**d**) Hardness ***H***.

**Figure 4:**
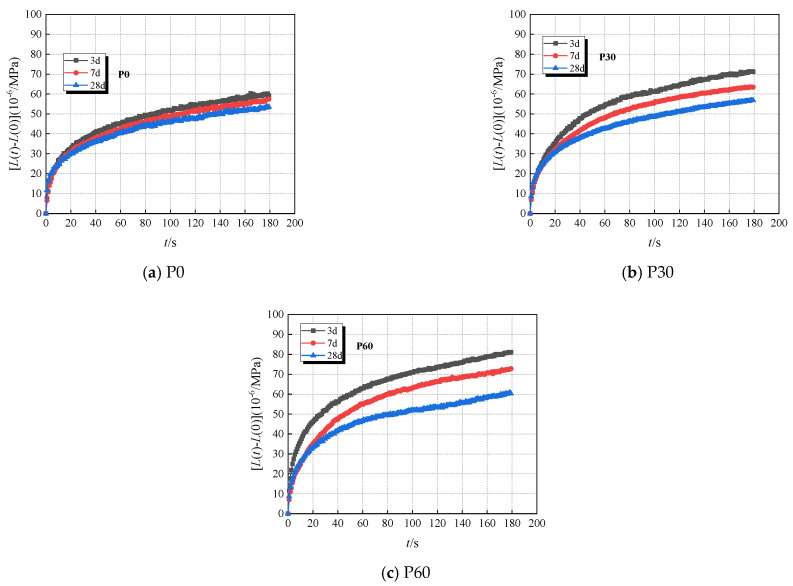
Variation of microscopic creep over time for different fly ash dosages. (**a**) P0, (**b**), P30 (**c**) p60.

**Figure 5:**
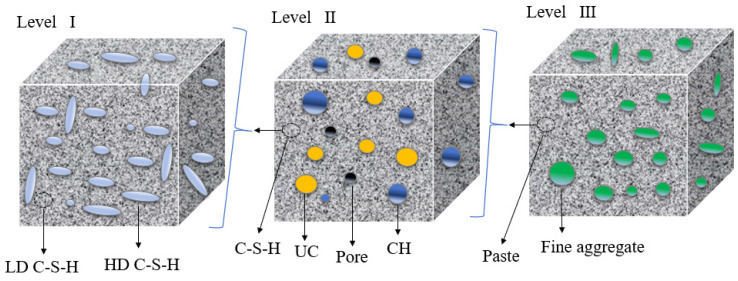
A diagrammatic sketch of LD C–S–H and HD C–S–H.

The results of ***M*** (the indentation modulus), ***H*** (hardness), ***A_C_***, ***L(t)-L(0)***, ***τ*** of the paste with 0%, 30%, and 60% fly ash are presented in Figure 3a–d at different test ages. The data reveal that both P60 and P30 specimens exhibit greater ***h_m_*** and ***A***_c_ values than those of P0 at a consistent test age; conversely, the ***h_m_*** and ***A***_c_ of P0, P30, and P60 at a constant fly-ash content diminished when the test age progresses. The trends observed in ***H*** and ***E*** were inversely related to those of ***h_m_*** and ***A***_c_. These micro-mechanical parameters signify the trend in the paste’s rheological properties with advancing test age when fly-ash concentrations change. Based on Equation (9), the micro-scale creep average value of hardened cement paste obtained from the nanoindentation experimental test results is shown in Figure 4. These results indicated that the specimens P60 and P30 exhibit more pronounced micro-scale creep than P0 at the same test age because of the fly ash. The results of the nanoindentation showed that the hardness of the cementitious paste developed slowly at equivalent ages because of the inclusion of extensive fly-ash quantities [17,50]. The incorporation of fly ash has an obvious influence on the hydration process of cementitious materials. The development of paste stiffness was also affected; the secondary reaction of fly ash was compensatory at a subsequent age [51]. So, the micro-strain of a paste containing high-volume fly ash (HVFA) showed lager value than that without fly ash. As the test age extended, a decreasing trend in the microscopic creep was observed, attributable to the increase of the past’s HD C–S–H gel content (LD C–S–H and HD C–S–H diagrammatic sketch as illustrated in Figure 5) [39]. Additionally, the paste’s HD C–S–H gel content growth and consolidation enhance the paste’s structural integrity, altering its rheological properties, increasing stiffness and decreasing its deformation ability.

The microscopic creep data obtained from experiments were subjected to a least square fit to determine the creep modulus ***C*** and characteristic time ***τ***. Table 5 provides parameters ***C*** and ***τ*** of the P0 group, the P30 group, and the P60 group from the microscopic creep, respectively. Both ***C*** and τ exhibited an increase trend with the age, corroborating the previous findings regarding the age period’s impact on microscopic creep evolution. Within the same age period, the experimental data revealed that the incorporation of fly ash resulted in the reduction of the values of ***C*** and ***τ***. This observation underscores that the addition of fly ash accelerates the rate of early creep development.

### 4.2. Tensile Creep of HVFAC Developments Depending on Age

#### 4.2.1. Effect of Fly Ash Dosages on the Tensile Specific Creep of HVAFC

**Figure 6:**
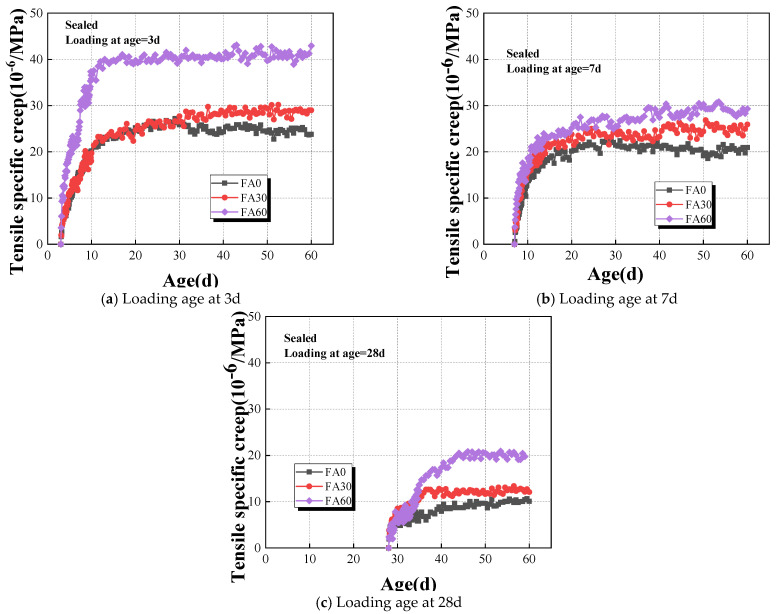
Effect of fly ash dosages on the tensile specific creep of HVAFC under sealed condition.

The results of tensile specific creep to HVAFC with different fly ash dosages under sealed conditions are illustrated in Figure 6. The results show that the early-age tensile specific creep of HVFAC is highly sensitive to the fly-ash dosage. For example, at the loading time continuing to 60 days, the specific creep of the FA30 and FA60 groups at the loading age of 3d is 1.18 and 1.79 times that of the FA0 group, respectively. It is due to the substitution of cement with fly ash, which slows down the hydration reaction of concrete, and the secondary reactions of the fly ash are delayed [40]. The strength and stiffness of FA60 at early ages are significantly reduced compared to that of FA30 at same ages, and the tensile specific creep of FA60 increased too. Compared with Fa30 and FA60, the tensile specific creep increased more pronouncedly at early ages when the fly-ash content increased. However, the tensile specific creep increased weakly at later loading ages because of the addition of fly ash, particularly at loading ages of 7d and 28d. The tensile specific creep values of concrete with different fly ash contents were closer to each other at later stage, such as at age of 45d or later. For instance, the tensile specific creep of the FA30 and FA60 groups is 1.24 and 1.40 times greater than that of the FA0 group at the loading time of 60 days with a loading age of 7d, respectively. Therefore, it can be asserted that the impact of the fly ash dosage on the initial stages of concrete development is substantial, whereas the influence of fly ash-containing concrete in the mature phase gradually wanes and ultimately tends towards stabilization.

#### 4.2.2. Effect of Loading Age on the Tensile Specific Creep of HVAFC

**Figure 7:**
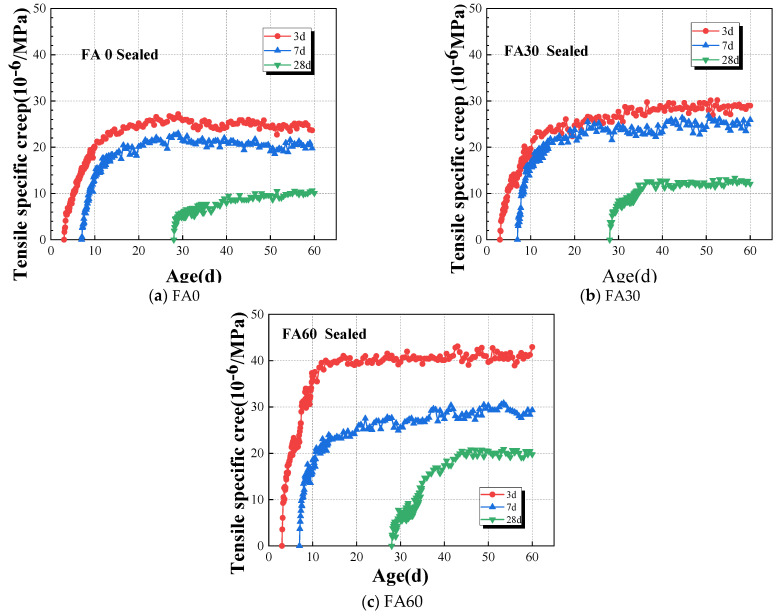
Effect of loading age on tensile specific creep of HVAFC under sealed condition.

Obviously, the strength and stiffness of the concrete are all in the process of development, and concrete’s ability to resist external loads increases at early ages. Thus, it cannot be ignored that the loading age influences the creep behavior law of concrete at early ages. The start point of loading age was defined as the point at which the creep specimens were initially subjected to the application of load in this study. The results of loading age on the tensile specific creep of HVFAC under sealed conditions are shown in Figure 7. It was evident that early concrete tensile specific creep was particularly sensitive to the factor of loading age. This sensitivity arises because the microstructure of early-stage concrete is not fully developed, and the ongoing hydration and formation of micro-cracks render the material more susceptible to continuous loading [50,52]. As the concrete matures, its microstructure becomes more stable, resulting in lesser creep when loaded at later stages. For instance, the specific creep for the FA60 group loaded at 3d was 1.47 and 2.17 times that observed for loading at 7d and 28d, respectively, while the specific creep for the FA0 group loaded at 3d was 1.14 and 2.36 times that of 7d and 28d loading, respectively. The influence of loading age on creep development of concrete was more pronounced in groups with higher cement replacement by an equal mass of fly ash. The reason for that was the retardation of the concrete’s hydration reaction by fly ash.

## 5. Discussion

### 5.1. The Role of Paste’s Rheological Properties

**Figure 8:**
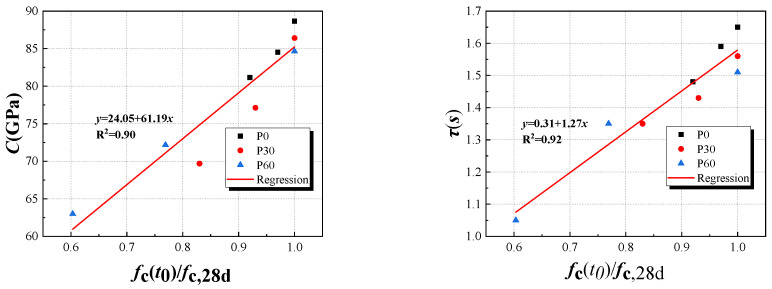
Correlation between creep modulus *C*, characteristic time *τ*, and $R_{c}\left(t_{0}\right)/R_{c,28d}$.

**Figure 9:**
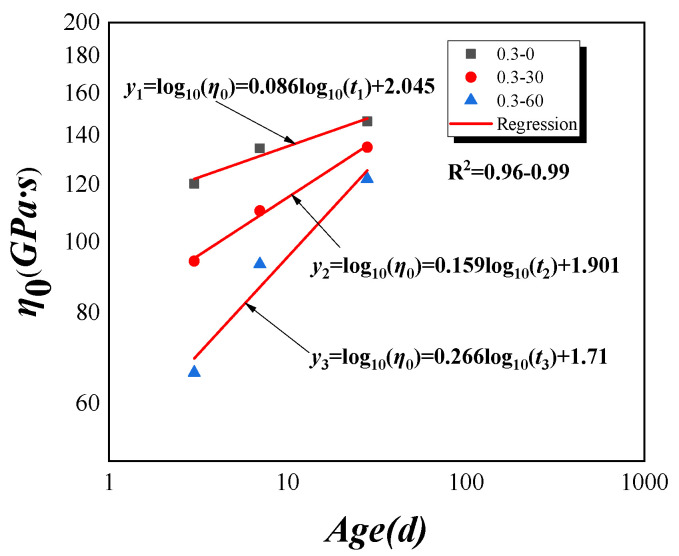
Double logarithmic coordinate curve of initial cohesion factor vs. ages.

In order to expose the influences of paste’s rheological characteristic on tensile creep of HVFAC at early ages, based on the physical significance of the ZC model parameter ***r*** shown in Equation (4), the initial viscosity coefficient of HVFA cementitious paste can be obtained under the same paste mix conditions in HVFAC, and the parameters from the micro-scale are shown in Table 5. The correlation between the creep modulus ***C***, the characteristic time ***τ***, and the relative compressive strength of the paste $R_{c}\left(t_{0}\right)/R_{c,28d}$ are shown in Figure 8. The double logarithmic plot and fitting results of the initial viscosity coefficient versus ages are shown in Figure 9. The initial viscosity coefficient and age exhibit a linear relationship in the double logarithmic coordinates, and this phenomenon is also observed in other studies [53,54]. The initial viscosity coefficients at 3d, 7d, and 28d for the groups P0, P30, and P60 are 120.12 GPa·s, 134.37 GPa·s, 146.27 GPa·s, 94.08 GPa·s, 110.30 GPa·s, 134.80 GPa·s, and 66.14 GPa·s, 93.16 GPa·s, 121.92 GPa·s, respectively. These results indicated that the initial viscosity coefficients of the groups P0, P30, and P60 increase as the testing age progresses, while the fly ash reduces the initial viscosity coefficient of the paste. A higher initial viscosity in concrete suggests a stronger resistance to deformation at the initial state, typically implying higher internal friction and lower fluidity. Additionally, the initial viscosity coefficient affects the deformation rate during the creep process; higher initial viscosity may slow down the creep rate as internal viscous resistance hampers the movement and adjustment of the microstructure. Moreover, the rate of development of the initial viscosity accelerates with an increase in fly ash content. This is because fly ash, due to its lower reactivity, does not participate in the hydration reaction in the early stages, resulting in a lower initial viscosity coefficient. However, as the age progresses, secondary reactions between fly ash particles and cement hydration products occur, with fly ash particles gradually hydrating and their bonding capacity increasing, thereby enhancing the viscosity of concrete. The C–S–H gel progressively fills these pores, reducing their size and consequently causing a gradual increase in the initial viscosity coefficient [53].

.

### 5.2. The ZC Model Parameters Based on the Rheological Properties of Paste

**Figure 10:**
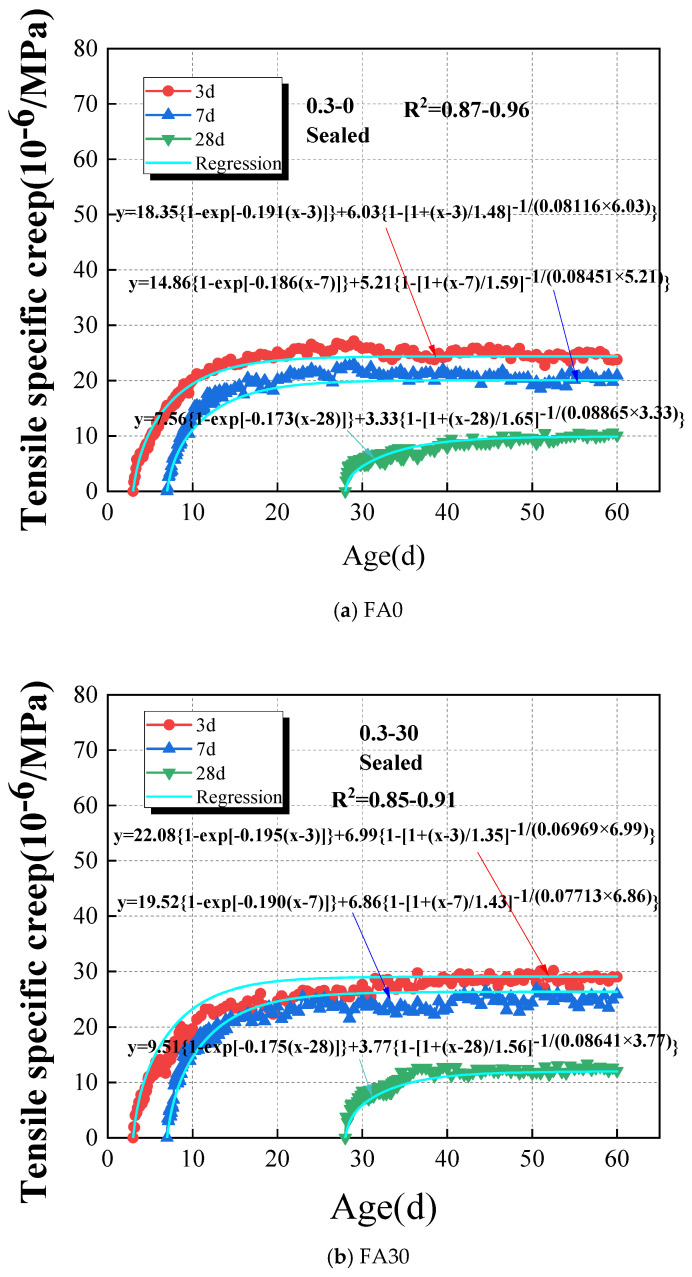

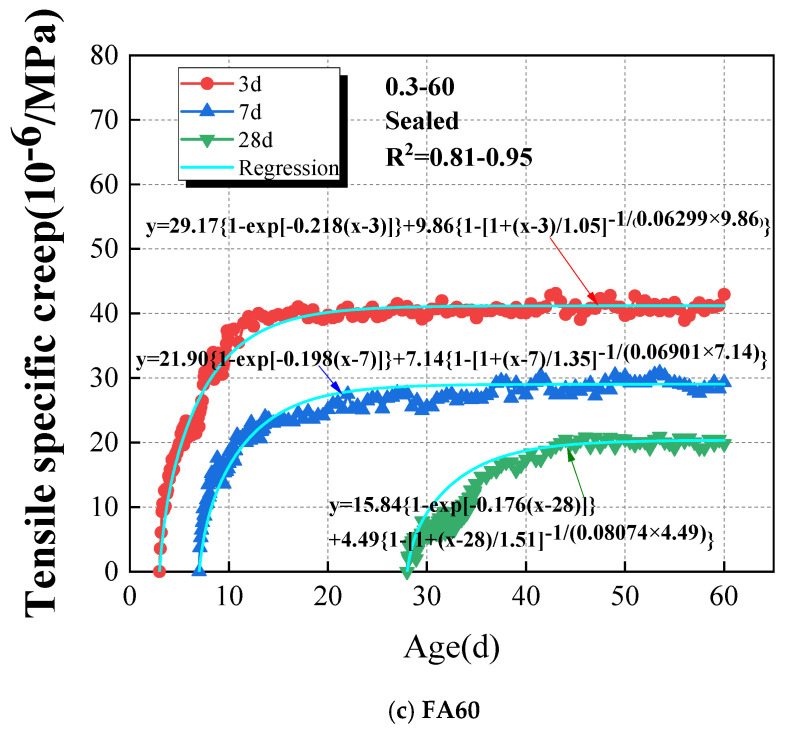
Tensile specific creep experimental values and ZC model predictions.

The comparative results of the experimentally obtained specific creep values and the model regression values for the FA0, FA30, and FA60 groups at loading ages of 3d, 7d, and 28d are illustrated in Figure 10. At the loading ages of 3d, 7d, and 28d, the basic tensile creep of HVFAC is consistently observed in the order of FA60 > FA30 > FA0. Previous research indicates that the incorporation of fly ash improves the microstructure of concrete; however, replacing cement with an equal mass of fly ash reduces the early hydration of concrete, leading to a comparatively looser structure of the hardened cement paste consistent with the findings of Hashmi et al. [54]. This is may be attributed to the pozzolanic reactions initiated by the addition of fly ash, which typically begin to occur between 3 to 14 days after the beginning of the hydration reaction of cement materials [27]. The tensile creep of concrete with the fly ash decreases when the loading ages are delayed. This phenomenon is obviously when the concrete deformation under tensile stress is constrained by the development of concrete stiffness. The elastic modulus of early concrete increased rapidly during the initial stage and then gradually levelled off, and this results in a continuous decrease in tensile creep with later loading ages or load-holding time following. This trend of tensile creep decreasing over time is typically considered a result of concrete aging and can be explained by the consolidation theory [55]. The effects of fly ash dosages and loading age on the creep properties of concrete are consistent with the micro-creep properties of the cementitious paste. The tensile specific creep values derived from the ZC model based on nanoindentation data closely match those obtained from experiments, and the results are shown in Figure 10.

The three key parameters of the ZC model ***(φ, 1/χφ, 1/(E_V_*** ***+ E_H_))*** were obtained from the multivariate nonlinear fit, and they are listed in Table 6. The parameter ***φ*** represents the coefficient influencing the rate of increase in the viscosity coefficient; the parameter ***1/χφ*** corresponds to the final specific creep of the Maxwell body; and the parameter ***1/(E_V_ + E_H_)*** denotes the final specific creep common to both the Kelvin body and the spring body. These three parameters are presented in Table 6. The parameter ***φ*** decreases as the loading age increases, indicating that the creep rate develops more rapidly in the early stages. Similarly, the parameters ***1/χφ*** and ***1/(E_V_ + E_H_)*** also decrease with the prolongation of loading age, aligning with the physicochemical implications of these parameters in the model. This may be because the later the loading age, the more complete the hydration reaction of the concrete, making the internal structure of the concrete denser and exhibiting a suppressive effect on the creep properties. Additionally, the incorporation of fly ash in concrete delays the hydration reaction, slows the early strength development, and leaves more free gel water available for movement, which can enhance the development of creep. In the model, this phenomenon corresponds to an increase in ***1/χφ*** and ***1/(E_V_ + E_H_)*** with the fly ash content increase.

### 5.3. The Relations Between the Parameters of ZC Model with Compressive Strength at 28d Ages

**Figure 11:**
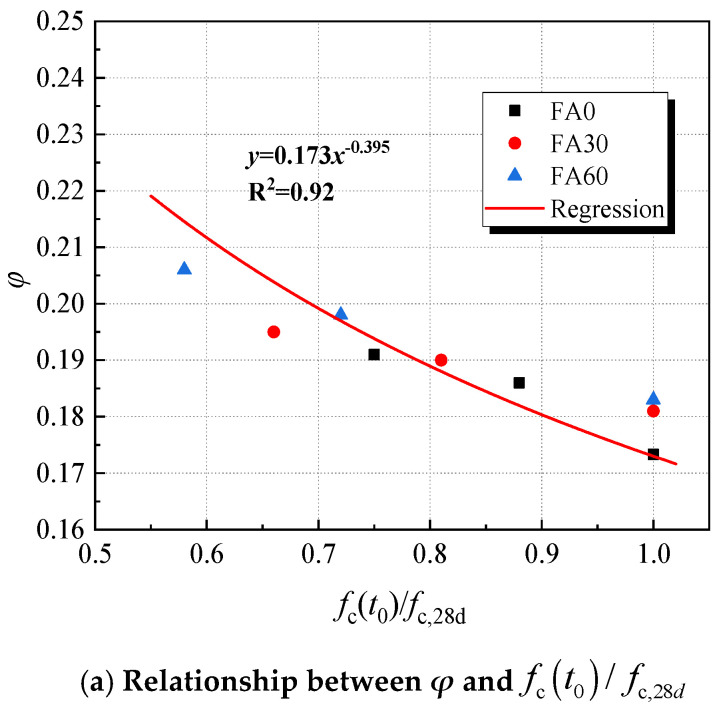

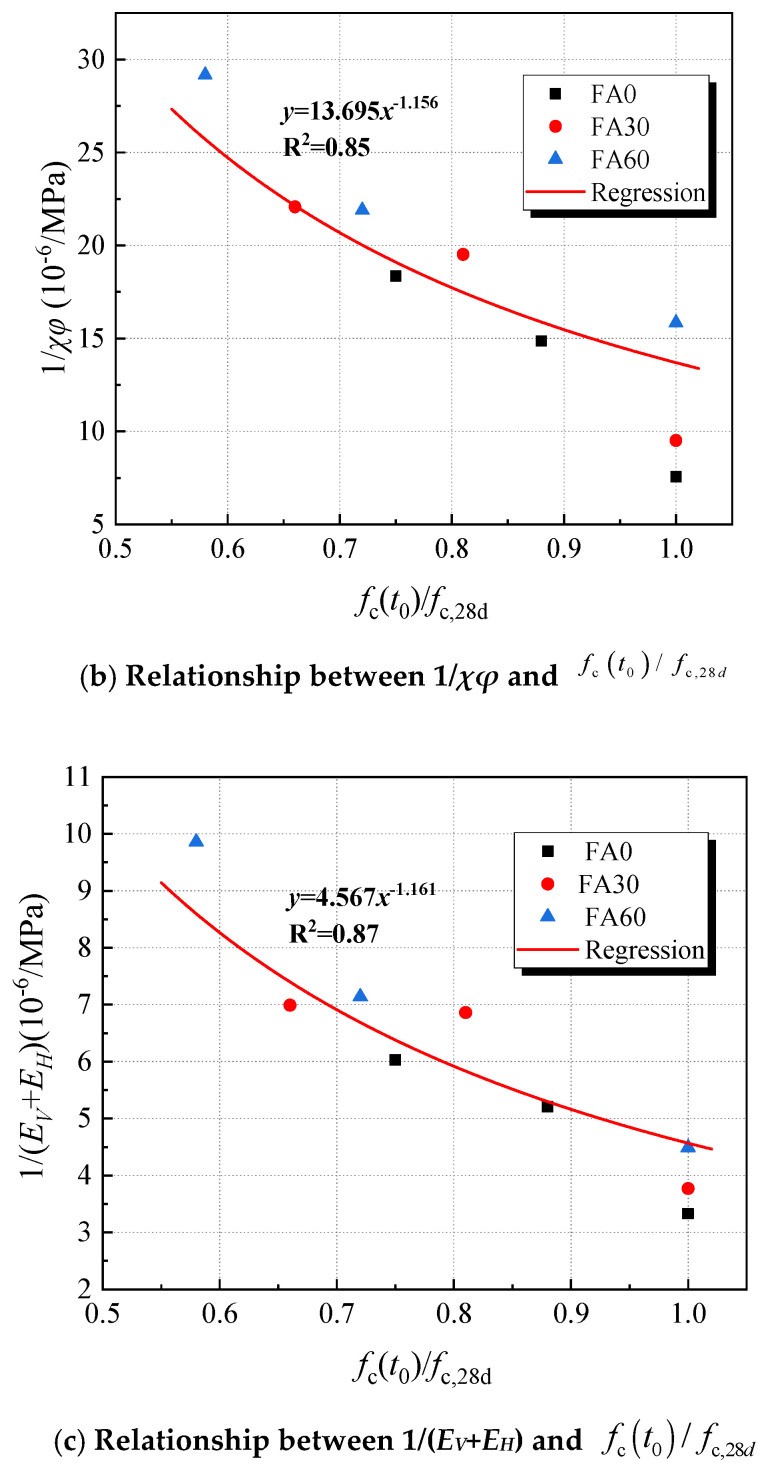
Relationship between the parameters in the model and the relative compressive strength.

The ZC model introduces the relative compressive strength ***f_c_(t_0_)/f_c,_*_28*d*_**, representing the ratio of HVFAC’s compressive strength at ***t_0_*** to its 28d compressive strength. This ratio is intimately linked to the hydration level at ***t_0_*** and influences the early tensile creep of HVFAC. Accordingly, this study conducts a fitting analysis of ***E_t,_*_28*d*_*/(E_V_ + E_H_)***, ***E_t,_*_28*d*_*/χφ****, **φ***, and ***f*_c_(*t*_0_)/*f*_c,28d_**, with the results displayed in Figure 11. Simultaneously, the correlation analysis of ***C*** and ***τ*** with the test age presented in Table 5 shows that the correlation aligns well with the linear relation, as depicted in Figure 8. Based on the analysis of the correlation and the changing patterns of each parameter, it is possible to further refine the prediction expression for the ZC model. This refinement offers a valuable reference for predicting the tensile creep of HVFAC. Based on the experimental results, the dispersion of the regression curve equations is within the acceptable range, and it is reasonable to use the development of each parameter in the relative compressive strength prediction model. The relationship between each parameter and the relative compressive strength is shown in Equations (16)–(18):

(16) $$\varphi =0.173{\left[f_{c}\left(t_{0}\right)/f_{c,28d}\right]}^{-0.395}$$

(17) $$\frac{1}{\chi \varphi }=13.695{\left[f_{c}\left(t_{0}\right)/f_{c,28d}\right]}^{-1.156}$$

(18) $$\frac{1}{E_{V}+E_{H}}=4.567{\left[f_{c}\left(t_{0}\right)/f_{c,28d}\right]}^{-1.161}$$

Based on the relationship of each parameter with the relative compressive strengths of HVFA cementitious net paste and HVFAC, respectively, in Figure 8 and Equation (15), a predictive expression of the tensile creep of HVFAC based on the rheological properties of the paste can be obtained:

(19) $$\begin{array}{l} C(t,t_{0})=13.695{\left(f_{c}\left(t_{0}\right)/f_{c,28d}\right)}^{-1.156}\left\{1-\exp[-0.173{\left(f_{c}\left(t_{0}\right)/f_{c,28d}\right)}^{-0.395}(t-t_{0})]\right\}\\ +4.567{\left(f_{c}\left(t_{0}\right)/f_{c,28d}\right)}^{-1.161}\left\{1-{\left[1+\frac{1}{0.31+1.27\left(R_{c}\left(t_{0}\right)/R_{c,28d}\right)}\left(t-t_{0}\right)\right]}^{-\frac{1}{4.567{\left(f_{c}\left(t_{0}\right)/f_{c,28d}\right)}^{-1.161}\left[24.05+61.19\left(R_{c}\left(t_{0}\right)/R_{c,28d}\right)\right]}}\right\} \end{array}$$

where ***f_c_(t_0_)/f_c,28d_*** is the relative compressive strength of concrete, and ***R_c_(t_0_)/R_c,28d_*** is the relative compressive strength of paste.

## 6. Conclusions

1. The experimental results of nanoindentation showed that the maximum indentation depth (***h_m_***) and the contact radius (***A_c_***) of paste decreased when the test age was delayed. The values of parameters ***h_m_*** and ***A_c_*** increased with the inclusion of fly ash, and the results showed that the microscopic creep of paste with a high-volume fly ash admixture converges rapidly as the test age increases. The creep modulus (***C***) and the characteristic time (***τ***) both increased when the test time was delayed, and it could be inferred that the rate of creep development decelerated.
2. Under the conditions of HVFAC with the same mass fraction of cementitious materials, the substantial incorporation of fly ash could facilitate the development of creep. At loading ages of 3d, 7d, and 28d, the specific creep of the FA60 group at 60d was 2.19, 1.54, and 3.66 times greater than that of the FA0 group, respectively.
3. The fly ash contributed to the development of microscopic creep in paste at the same test age. The microscopic creep of the FA60 group tended to converge rapidly with the extension of the test age. The correlation analysis between parameters ***E_t_***,_28d_/(***E_V_ + E_H_***), ***E_t_***,_28d_/***χφ***, ***φ***, and the relative compressive strength ***f_c_(t_0_)/f_c,28d_***, ***C***, and ***τ*** with the test age indicated a good fit with the power function. Based on the combination of the paste’s parameters of the rheological properties and the parameters of the ZC model, a predictive expression for predicting the development of HVFAC tensile creep over time can be established.
4. Within the same age period, the experimental data revealed that the incorporation of fly ash resulted in the values of the creep modulus *C* and characteristic time *τ* reduced. The effects of fly ash dosages and loading age on the creep properties of concrete are consistent with the micro-creep properties of the cementitious paste. The tensile specific creep values derived from the ZC model based on the nanoindentation data closely match those obtained from experiments.

## Figures and Tables

**Table 1:** Chemical compositions of fly ash and cement.

Material	Mass Fraction %								
	CaO	SiO_2_	Al_2_O_3_	MgO	Fe_2_O_3_	SO_3_	K_2_O	Na_2_O	Others
Cement	62.04	22.07	4.23	4.01	3.04	2.71	0.762	0.392	0.746
FA	4.43	51.63	33.98	1.16	4.40	0.260	0.905	0.888	2.347

**Table 2:** Proportions of paste.

№	W/(Cement+Fly Ash)	Fly Ash/*wt*%	Cement/*wt*%
P0	0.3	0	100
P30		30	70
P60		60	40

**Table 3:** Proportions of HVFAC (kg/m^3^).

№	W/(Cement+Fly Ash)	Cement	FA	Water	Fine Aggregate	Coarse Aggregate	Superplasticizer
FA0	0.3	600	0	180	118	481	1.32
FA30		420	180	180	118	481	1.44
FA60		240	360	180	118	481	1.80

**Table 4:** Mechanical Properties of HVFAC.

№	Age of Testing/d	Compressive Strength/MPa	Splitting Tensile Strength/MPa	Tensile Elastic Modulus/GPa
FA0	3	56.04	5.60	33.40
	7	65.71	6.31	38.00
	28	74.84	6.40	38.25
FA30	3	39.31	3.40	29.10
	7	48.31	4.53	33.65
	28	60.00	5.24	35.95
FA60	3	25.62	2.50	19.75
	7	32.02	3.20	26.10
	28	44.24	3.94	28.50

**Table 5:** Parameters of microscopic creep.

№	Age of Testing/d	The Creep Modulus/GPa	*τ*/s
P0	3	81.16	1.48
	7	84.51	1.59
	28	88.65	1.65
P30	3	69.69	1.35
	7	77.13	1.43
	28	86.41	1.56
P60	3	62.99	1.05
	7	69.01	1.35
	28	80.74	1.51

**Table 6:** Parameters of the ZC model for HVFAC based on the paste’s rheological properties.

№	Loading Age/d	*φ*	1/*χφ*/10^−6^·MPa^−1^	1/(*E_V_* + *E_H_*)/10^−6^·MPa^−1^
FA0	3	0.191	18.35	6.03
	7	0.186	14.86	5.21
	28	0.173	7.56	3.33
FA30	3	0.195	22.08	6.99
	7	0.190	19.52	6.86
	28	0.175	9.51	3.77
FA60	3	0.218	29.17	9.86
	7	0.198	21.90	7.14
	28	0.176	15.84	4.49

## Data Availability

The data that support the findings of this study are available from the corresponding author upon reasonable request
